# Biochemical and Behavioral Responses in the Killer Shrimp *Dikerogammarus villosus* Following Acute Exposure to Thiacloprid and Calypso®

**DOI:** 10.1007/s00244-025-01130-z

**Published:** 2025-05-16

**Authors:** Dávid Somogyvári, Mária Mörtl, Anna Farkas, András Székács, János Győri

**Affiliations:** 1 Ecophysiological and Environmental Toxicological Research Group, Balaton Limnological Research Institute, Hungarian Research Network, Klebelsberg Kuno U. 3., Tihany, 8237 Hungary; 2 National Laboratory for Water Science and Water Security, HUN-REN Balaton Limnological Research Institute, Klebelsberg Kuno U. 3., Tihany, 8237 Hungary; 3 Agro-Environmental Research Centre, Institute of Environmental Sciences, Hungarian University of Agriculture and Life Sciences, Páter Károly U. 1., Gödöllő, 2100 Hungary; 4 Agrotechnology National Laboratory, Páter Károly U. 1., Gödöllő, 2100 Hungary

## Abstract

Neonicotinoids are insecticides that are used globally and can persist in soil and surface water, posing a threat to ecosystems. In this study, we exposed the invasive freshwater amphipod *Dikerogammarus villosus* to environmentally relevant and relatively high concentrations of thiacloprid, a widely used agricultural neonicotinoid active ingredient and its commercial form Calypso® for two days. The acute effects were investigated at the behavioral (immobility time) and biochemical [glutathione S-transferase (GST) and acetylcholine esterase (AChE) activity] levels. Calypso® concentrations of 10 µg/l and 100 µg/l a significantly increased the immobility time, while thiacloprid exerted such an effect only at 100 µg/l. The GST enzyme activity did not change in the thiacloprid-treated groups; however, the 10 µg/l and 100 µg/l Calypso® concentrations significantly increased the GST activity. All Calypso® concentrations significantly decreased AChE activity until the highest Calypso® concentration was reached, and an interesting outcome was the ‘U-shaped dynamics’ of AChE activity. In contrast, thiacloprid had no significant blocking effect on AChE activity at any of the concentrations tested. Neonicotinoid insecticides are neurotoxins that selectively target nicotinic acetylcholine receptors in the insect central nervous system. However, their widespread use has a growing impact on nontarget animals. This study confirms the risk of neonicotinoids to aquatic invertebrates by providing evidence that neonicotinoids can also affect both behavioral and biochemical processes in *D. villosus*.

Today, large amounts of various synthetic chemicals are released into the environment, including certain pesticides, in large quantities, contributing to chemical pressure on our ecosystems, including aquatic habitats. Among these pesticides, the presence of systemic insecticides from the neonicotinoid class is particularly worrying (Goulson 2013), which are detectable at low concentrations in soil, water and sediment due to their high water solubility (Sánchez-Bayo et al. 2016; Stehle et al. 2023).

Neonicotinoid-based insecticides were developed in the 1990s as alternatives to broad-spectrum insecticides. Bayer CropScience marketed imidacloprid (IMI), the first molecule in the family in 1991, launching the first generation of neonicotinoids. With the emergence of additional molecules, these neurotoxins became the most widely used products in agriculture (Jeschke et al. 2011), accounting by 2016 for almost 20–30% of the total insecticide market. The ease of use and effectiveness in controlling invertebrate pests are the main drivers of their growth. Neonicotinoids imitate the action of acetylcholine (Tomizawa and Casida 2005), a key stimulatory neurotransmitter of the central nervous system (CNS). Their primary mechanism of action is their strong binding to the postsynaptic nicotinic acetylcholine receptors (nAChRs) of the insect brain, which have shown a more selective pharmacological/toxicological profile in arthropods than do vertebrate receptors (Tomizawa 2013).

Since the mid-2000s, several studies have focused on the possible negative effects of the most widely used neonicotinoids on nontarget organisms (Thompson et al. 2020). As a result, active ingredients (AIs), including clothianidin (CLO), IMI and thiamethoxam (TMX), were banned as plant protection products in the EU in 2018. These three neonicotinoids were restricted to use in seed coating materials in 2013; thus, another neonicotinoid-type pesticide, thiacloprid (TIA), was applied for the treatment of corn seeds. The European Commission came to a decision in 2020 not to renew the approval of TIA. The authorization of acetamiprid (ACE) was renewed in 2018 until 2033, as its adverse effects seem less serious than those of the abovementioned ingredients. Some restrictions were also introduced by the US EPA to protect bees and other pollinators from neonicotinoid exposure. However, dockets for all neonicotinoids are still available, and the completion of risk assessments for neonicotinoids was postponed to 2025 (EPA 2024). The registrant voluntarily canceled the renewal of TIA. Notably, more than 120 countries worldwide still use neonicotinoids.

While the role of neonicotinoids in the mass mortality of pollinators has been the focus of intense research, data available for aquatic nontarget organisms, including mollusks, cladocerans, amphipods, crustaceans and amphibians (Finnegan et al. 2018; Rico et al. 2018; Ewere et al. 2021; Kowall et al. 2023; Jourdan et al. 2024; Lozano et al. 2024; Oliveira et al. 2025; Lin et al. 2025), are still limited. The wide sensitivity range within and between groups highlights the need for testing in a variety of aquatic invertebrates (Raby et al. 2018a), not only by testing typical model species (Raby et al. 2018b), to better understand the underlying mechanisms of neonicotinoid toxicity and thus potential risks.

Despite several studies indicating that many aquatic invertebrates may be sensitive to neonicotinoids, most of the literature is based on the assessment of IMI (Mora-Gutiérrez et al. 2021) and comparative data on other neonicotinoids are scarce (Thunnissen et al. 2022). Furthermore, laboratory studies typically use pure AIs to investigate the cellular and molecular mechanisms underlying general toxicity, but in the field, agrochemicals, including neonicotinoids, are used in formulations of complex mixtures of chemicals. Pesticides, including insecticides, are often used in practice as mixtures containing the AI together with formulating additives, called adjuvants, which are added to modify the physicochemical properties, stability or penetration of the AI. These substances can affect both target organisms, such as pest insects, and nontarget organisms (Takács et al. 2017), including beneficial insects, e.g., pollinators. Comparative investigations have shown that, compared with pure ingredients, formulated products often induce stronger effects, resulting in greater toxicity (Mörtl et al. 2020).

Hence, in the present study, we analyzed the insecticide AI TIA and one of its commercial formulations, Calypso®, to determine whether they produce similar or different responses on the killer shrimp *Dikerogammarus villosus*. The neonicotinoid was selected because of its widespread use as a pesticide in both intensive crop production and domestic use. Its moderate water solubility (185 mg/l at 20 °C) and the potential for runoff from agricultural fields contribute to its presence in surface waters, where it can reach concentrations that pose risks to aquatic organisms (Sánchez-Bayo and Hyne 2014; Berens et al. 2021; Raths et al. 2023; Ma et al. 2025). *D. villosus* is the ideal choice for aquatic monitoring studies due to its ecological importance for detritus decomposition, trophic transfer of nutrients and contaminants and widespread occurrence (Jermacz and Kobak 2018). It is also a typical nontarget organism and can take up contaminants through the gills (respiration) or their diet (i.e., contaminated leaves). As a result of its aggressive expansion, the number of native shrimp species has declined dramatically in Lake Balaton; however, *D. villosus* forms abundant populations. As previously demonstrated, *D. villosus* is two orders of magnitude more sensitive to the neonicotinoid (CLO) based formulated insecticide product Apacs® 50 WG than the widely used ecotoxicological model species *D. magna* (Somogyvári et al. 2020)*.* It was shown that acute exposure to two neonicotinoids, CLO and IMI, induces behavioral (immobility time) and biochemical alterations (AChE, GST) in the killer shrimp *D. villosus* (Somogyvári et al. 2022)*.* Behavioral response of native and invasive amphipods to neonicotinoid thiacloprid (33.92 μg/L) exposure were compared, and a slight decrease in activity (distance moved) of *D. villosus* was reported (Soose et al. 2024). The worst-case predicted environmental concentrations in surface waters resulting from the normal agricultural use of TIA reported by the manufacturer are 17.52 µg/l (Beketov and Liess 2008), but detected TIA concentrations in surface waters can vary significantly, with recorded levels ranging from 0.02 to 12.00 μg/l (Barmentlo et al. 2018). Since TIA is removed rapidly of the aqueous phase and transported to the sediment, the chance of detecting the maximum concentrations by grab sampling is low. We exposed acutely the animals to four nominal concentrations of TIA; 10, 30, 100 and 250 µg/l. These concentrations fall into the range of TIA toxicity among Crustaceans (Morrissey et al. 2015) with the lower concentration being observed in Dutch surface waters (Barmentlo et al. 2019). We investigated the effects of TIA on several endpoints (AchE and GST activity, and locomotion).

A comparison of TIA with Calypso® may explore the potential (synergistic or antagonistic) effect of formulating agents and additives, which has been previously reported for some plant protection products. An investigation of a formulated product allows a closer understanding of the field situations where pesticides (in the form of a mixture of chemicals) appear in the environment.

Biomarkers have been shown to be effective indicators of environmental contamination and are widely utilized to evaluate initial responses to various chemicals. The assessment of locomotor activity as a potential biomarker of exposure to environmental pollutants has been proposed for the monitoring of freshwater ecosystems (Wallace and Estephan 2004; Felten et al. 2008). Alterations in locomotion behavior (as swimming duration) is a sensitive indicator of depressed ability of this organism to escape from unfavorable environment or escape from predators. Decreased locomotor activity may impair the organism’s survival by negatively affecting also the feeding of organisms and is considered potent biomarker for neuronal disruption in aquatic organisms in general, as evidenced by previous studies (Little and Finger 1990; Scott and Sloman 2004; Bownik 2017). Since the oxidative stress enzyme glutathione S-transferase (GST) and the cholinergic enzyme acetylcholinesterase (AchE) have been measured previously as enzymatic biomarkers of exposure of crustaceans to neonicotinoids (Jemec et al. 2007; Butcherine et al. 2022), changes in their activity were investigated.

## Materials and Methods

### Chemicals

We used Pestanal-grade TIA (CAS No. 111988-49-9, Sigma-Aldrich) and its formulated insecticide product Calypso® 480 SC marketed by Bayer Crop Science Corp., Monheim am Rhein, Germany, containing 40.4% TIA as the AI. The appropriate volumes were added to the experimental plastic tanks to reach the desired nominal concentrations. Translucent polypropylene plastic containers manufactured without plasticizers for food storage were used as treatment vessels to ensure easier observation of test organisms, reducing in the meantime their disturbance as they could not observe motion in their external surroundings. In prior experiments, the incidence of chemical loss from the exposure media due to adhesion to the wall of containers was assessed and considered negligible, being below 5% of the preset nominal concentrations. On the basis of the recovery data, the desired exposure concentrations of TIA were constant during the 2-day exposure.

### Experimental Animals, Acute Treatments and Chemical Analysis

Our *D. villosus* culture originated from the littoral region of Lake Balaton has been maintained at the Balaton Limnological Research Institute (Tihany, Hungary) for several years. The specimens were kept in large holding tanks (with ~ 100 individuals/tank stocking density) containing 10 l of oxygenated ‘unfiltered Balaton water’ at a constant temperature of 20 °C (± 1 °C) under a light:dark regime of 16 h:8 h. The animals were fed on carrot (*Daucus carota* subsp. *sativus*) ad libitum two times a week. To ensure that the shrimp had places in the exposure tanks to hide, which is characteristic of their behavior, small (diameter: 10 mm) stones were placed into the holding tanks. All procedures were performed according to the protocols approved by the Scientific Committee of Animal Experimentation of the Balaton Limnological Research Institute (VE-I-001/01890-10/2013).

From our culture, adult (7 mm length) specimens were selected for acute neonicotinoid exposure. To investigate adaptive behavioral and biochemical responses, the animals (*n* = 10/experimental group) were placed together in 600 mL of ‘unfiltered Balaton water,’ pH (8.5–8.6), salinity (280–450 mg/l), dissolved oxygen (around 10 mg/l), conductivity (600–700 μS/cm) and redox potential (400–600 mV), in 700 ml plastic tanks and exposed to different concentrations of TIA or Calypso® for 2 days. The samples in the control group were kept in 600 ml of ‘unfiltered Balaton water.’ The 5 experimental groups (1 control and 4 treated) of TIA/Calypso® exposure contained *n* = 50 total animals per replicate. Three replicates were set up for the experimental groups. The animals were food deprived for 2 days before the treatment as well as during the entire exposure period as the administration of carrot-based diets has been demonstrated to confer a protective effect against potential cases of poisoning. This phenomenon pertains to a particular property of β-carotene in carrots, wherein it may function as an antioxidant within the animal's physiological system. Consequently, the physiological changes induced by the toxic substances, in this instance insecticides, do not accurately reflect the actual level of poisoning. To avoid this, animals were starved before the experiments. The exposure concentrations for TIA were 10, 30, 100 and 250 µg/l, while the Calypso® concentrations were set to ensure that the AI concentrations during the treatments were the same as those for the pure compound.

We used a static exposure system for 2 days of neonicotinoid exposure. The initial solutions tested in the experiments were analytically measured. At the end of the treatment, quantification was also performed following our previously published methods (Farkas et al. 2022). Briefly, we used a Younglin YL9100® HPLC system (YL Instruments, Anyang, Republic of Korea) equipped with a YL9150 autosampler (YL Instruments, Anyang, Republic of Korea). The AIs were separated on a C18 column (150 mm × 4.6 mm i.d., 5 µm) at 40 °C. UV signals were recorded at 252 and 269 nm. The eluent flow rate was 1.0 ml/min with isocratic elution for 10 min (65:35 = A:B eluents, A = 90% water:10% MeOH, B = MeOH). Exposure media of control treatments did not contain any interfering matrix component; therefore, the quantitation of AIs was performed based on external calibrations with standard dilutions within the 0.050–120 mg/l concentration range. The limit of detection (LOD) and the corresponding limit of quantification (LOQ) for the TIA standard were 30 µg/l and 90 µg/l, respectively. For exposures in the lower dosages (10 and 30 µg/l treatment), where the expected TIA concentrations fell below LOQ, the AI was extracted three times from 5 ml of sample by using 100 μl of methanol and 2 ml of dichloromethane. The lower layers were combined, the organic phase was evaporated to dryness and the residue was resolved in 500 µl of HPLC eluent used as mobile phase. Recoveries were determined in triplicates with spiked solutions containing 10 µg/l of TIA. Worthy of note that addition of the appropriate amounts from Calypso® was not so easy due to the viscous property of the formulated pesticide. Therefore, a stock solution was prepared by dilution of 100 µl of Calypso® to 100 ml and its exact concentration was determined. This stock was used for further dilutions and to set the same exposure levels, as for the pure TIA as an AI (10, 30, 100 and 250 μg/l). Similarly to the low levels of TIA standard solutions, the insecticide AI was extracted and the tenfold concentrated solutions were measured. Water samples collected were not immediately subjected to analysis or prepared prior to HPLC–UV measurement; they were frozen for storage and later analyzed.

### Behavioral Assays

Following our previously published protocol (Somogyvári et al. 2020), the animals from the control and treated groups were individually placed in 6-well plates (*n* = 10 animals/group/replicate). The immobilization time (locomotion activity) was investigated for 1.5 min via a video tracking system. The videos were analyzed with Fiji ImageJ 1.53q software (public domain). The program recorded the coordinates and automatically reconstructed the trajectories of the test animals swimming within the ring of the whole plate.

### Total Protein Content and Enzyme Activity

The total protein content, as well as the GST and AChE activities, was measured on the basis of our previous works (Győri et al. 2017; Zaller et al. 2021). Briefly, ten adult animals from each group (*n* = 10 animals/group/replicate, 3 replicates) were pooled and homogenized using a TissueLyser LT (Qiagen, Germantown, MD, USA) device in 200 µl of phosphate-buffered saline (0.1 M, pH = 7.4). After centrifugation (10,000 g for 25 min at 4 °C), the supernatant was used for the biochemical measurements. The total protein content was measured via the Bradford assay (#B6916, Sigma-Aldrich, Schnelldorf, Germany) in a 250 µl total volume containing 5 µl of supernatant.

The GST activity was investigated with a GST Assay Kit (#CS0410, Sigma-Aldrich, Schnelldorf, Germany) in a 200 µl total volume containing 20 µl of supernatant and 180 µl of phosphate buffer (0.1 M, pH 6.5) with 10 mM 1-chloro-2,4-dinitrobenzene (CDNB) and 10 mM *L*-glutathione (reduced form, GSH) as substrates. GSTs catalyze the conjugation of the substrate, forming a thioether that can be followed by increasing the absorbance at 340 nm at 25 °C for 3 min every 15 s.

Following the methods of Ellman (Ellman et al. 1961) adapted to 96-well microtiter plates (Hamers et al. 2000), AChE activity was measured in 250 µl total volume containing 25 µl of supernatant and 225 µl of sodium phosphate buffer (0.1 M, pH 7.2) with 0.5 mM dithiobis-(2-nitrobenzoate) (DTNB) (#D8130, Sigma-Aldrich, Schnelldorf, Germany) and 7.5 mM acetylthiocholine iodide (AcThI) (#01480, Sigma-Aldrich, Schnelldorf, Germany). The hydrolysis of AcThI by AChE produces a thiol group that reacts with DTNB, resulting in the development of a yellow color (thionitrobenzoate). The color intensity of the product was measured at 405 nm at 25 °C for 3 min every 15 s and was proportional to the enzyme activity.

All measurements were implemented using a Victor 3 plate reader (PerkinElmer, Springfield, IL, USA). The reaction mixture without samples was used as a blank. For GST activity, the results are expressed as micromoles of thioether produced per minute per milligram of protein. For AChE activity, the results are expressed as micromoles of hydrolyzed acetylcholine iodide produced per minute per milligram of protein.

### Data Analysis

The data were statistically analyzed and plotted with the OriginPro 2022 9.9.0.225 (Academic) software package (OriginLab Corp., Northampton, MA, USA). The normality of the dataset was tested using the Shapiro–Wilk test, and the homogeneity of variances between groups was tested using the Levene test. For both behavior and enzyme activity measurements, differences among control and treated groups were analyzed by one-way ANOVA followed by Tukey’s post hoc test (*p* < 0.05). Mean differences were considered significant at *p* < 0.01 (**) and *p* < 0.05 (*), and asterisk(s) are shown in graphical representations.

## Results

### Analytical Measurements

Average recovery for spiked solutions containing TIA at the lowest level (0.010 mg/l) was 94 ± 4% and 95 ± 4% for TIA and Calypso®, respectively. No neonicotinoids were detected in the control water samples. The concentration of the stock solution, prepared from Calypso®, was checked. Instead of nominal concentration of 48 g/l for TIA, 61.4 g/l was obtained. Further dilutions were calculated accordingly to set the same exposure levels as for pure TIA. Analytical results, obtained for water samples collected after the test, are summarized in Table 1. There were no significant differences between the initial and final TIA contents of water samples following the 48 h exposures. Concentrations ranged between 93.9% and 101.5% compared to the nominal value, and only for the lowest exposure level (10 µg/l) was a 7% decline observed after the test for both TIA and Calypso® solutions. Recovery values measured in the spent exposure media recorded postexposures and summarized in Table 1 also demonstrated that no material losses of occurred during the experiments due to adhesion of the chemicals to the wall of the plastic containers used. Based on the data of Table 1, the concentrations were constant during 2-day exposure.

**Table 1:** Analytical data for test solutions with the active ingredient alone and in the tests with the pesticide formulation. Water samples were collected before (upper values) and after (lower values) the test

	TIA in standard solution (μg/l)				TIA in Calypso® (μg/l)			
	10*	30*	100	250	10*	30*	100	250
A	10.49 9.78	29.1 30.2	97.0 95.8	254.2 250.3	9.67 9.14	30.3 29.2	95.9 99.2	246.5 249.9
B	9.62/ 9.36	31.2 28.9	102.4 96.5	245.9 247.9	10.51 9.55	28.2 29.8	102.0 97.6	252.3 253.2
C	10.17 9.03	28.7 28.6	96.5 101.0	252.4 253.4	10.28 9.83	28.3 27.9	95.4 94.6	247.1 245.4
Average ± SD	10.09 ± 0.44 9.39 ± 0.38	29.7 ± 1.32 29.2 ± 0.9	98.6 ± 3.26 97.7 ± 2.8	249.2 ± 4.61 250.5 ± 2.8	10.15 ± 0.43 9.51 ± 0.35	28.9 ± 1.2 29.0 ± 1.0	97.8 ± 3.7 97.1 ± 2.3	249.7 ± 3.7 249.5 ± 3.9
RSD%	4.4% 4.0%	4.4% 3.0%	3.3% 2.9%	1.9% 1.1%	4.3% 3.7%	4.1% 3.5%	3.8% 2.4%	1.5% 1.6%

*These low levels are below the limit of quantitation (LOQ); therefore, these solutions were extracted, the tenfold concentrated solutions (about 100 or 300 μg/l) were measured and these are the calculated levels

### Behavioral Alterations of *D. Villosus* Exposed to Neonicotinoid Compounds

**Fig. 1:**
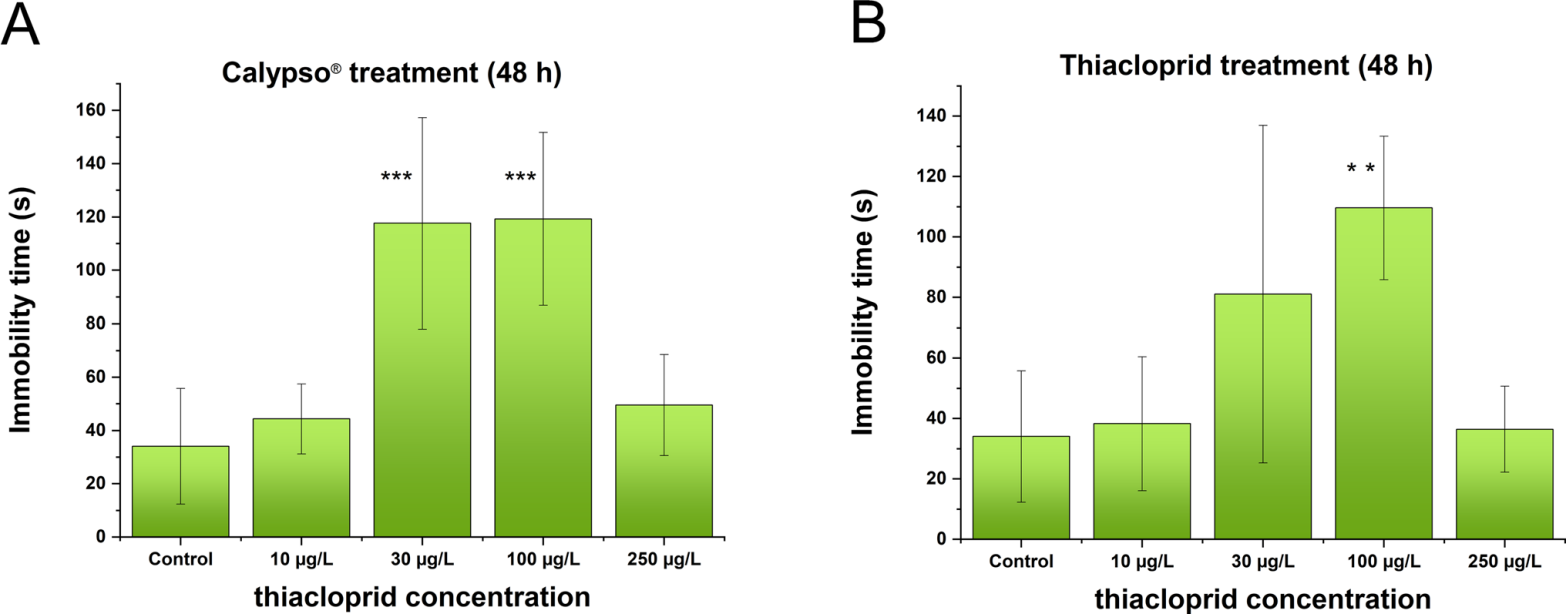
Effects of Calypso® (**A**) and thiacloprid (**B**) on locomotor activity—immobility time of *D. villosus*. Concentration values on the abscissa for Calypso® are nominal concentrations calculated from the composition of the formulation reported by the manufacturer, and for thiacloprid are nominal concentrations of the compound in its solutions applied in the test. Each bar represents the mean ± SD (*n* = 10 animals/group/replicate). Compared with that in the control group, a significantly increased duration of immobility was observed in the two groups treated with 10 µg/l 100 µg/l Calypso® (*p* < 0.001) (**A**), whereas a significantly increased immobility time was measured in the 100 µg/l thiacloprid (*p* < 0.05) treated group only (**B**)

The effects of Calypso® exposure on locomotor activity are shown in Fig. 1. The immobility time (Fig. 1A.) was significantly changed due to short-term (48 h) Calypso® exposure. Specifically, the duration of immobility was significantly greater in the 30 and 100 µg/l groups than in the control group (118 ± 5 s vs 33 ± 3 s).

The effects of TIA exposure at the behavioral level are presented in Fig. 1B. Compared to the control, only the 100 µg/l concentration significantly increased the immobility time (Fig. 1B).

### Changes in the GST and AChE activities of *D. villosus* Exposed to Neonicotinoid Compounds

**Fig. 2:**
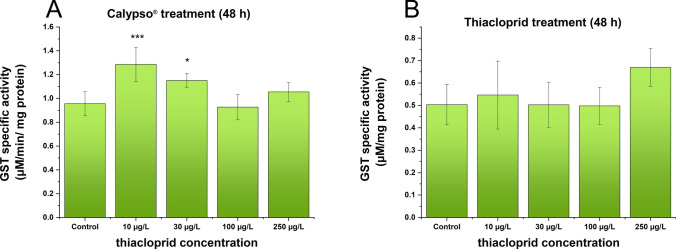
Changes in GST levels in *D. villosus* after Calypso® (**A**) and thiacloprid (**B**) treatments. Concentration values on the abscissa for Calypso® are nominal concentrations calculated from the composition of the formulation reported by the manufacturer, and for thiacloprid are nominal concentrations of the compound in its solutions applied in the test. Each bar represents the mean ± SD (*n* = 10 animals/group/replicate). Compared with the control, significantly increased GST activity was observed in the 10 (*p* < 0.001) and 30 µg/l (*p* < 0.05) Calypso®-treated groups (**A**). No significant differences were detected between the control and thiacloprid-treated groups (**B**)

The effects of two forms (pure AI and commercial product) of neonicotinoids on GST activity are shown in Fig. 2. There was an observable increase in the GST activity of *D. villosus* that were exposed to neonicotinoids in both cases. In the case of Calypso® exposure, there was a significant increase in GST activity at 10 and 30 µg/l compared with the activity recorded in the control groups (Fig. 2A). In the case of TIA exposure, there was no significant difference between the control group and the groups treated with TIA (Fig. 2B).

**Fig. 3:**
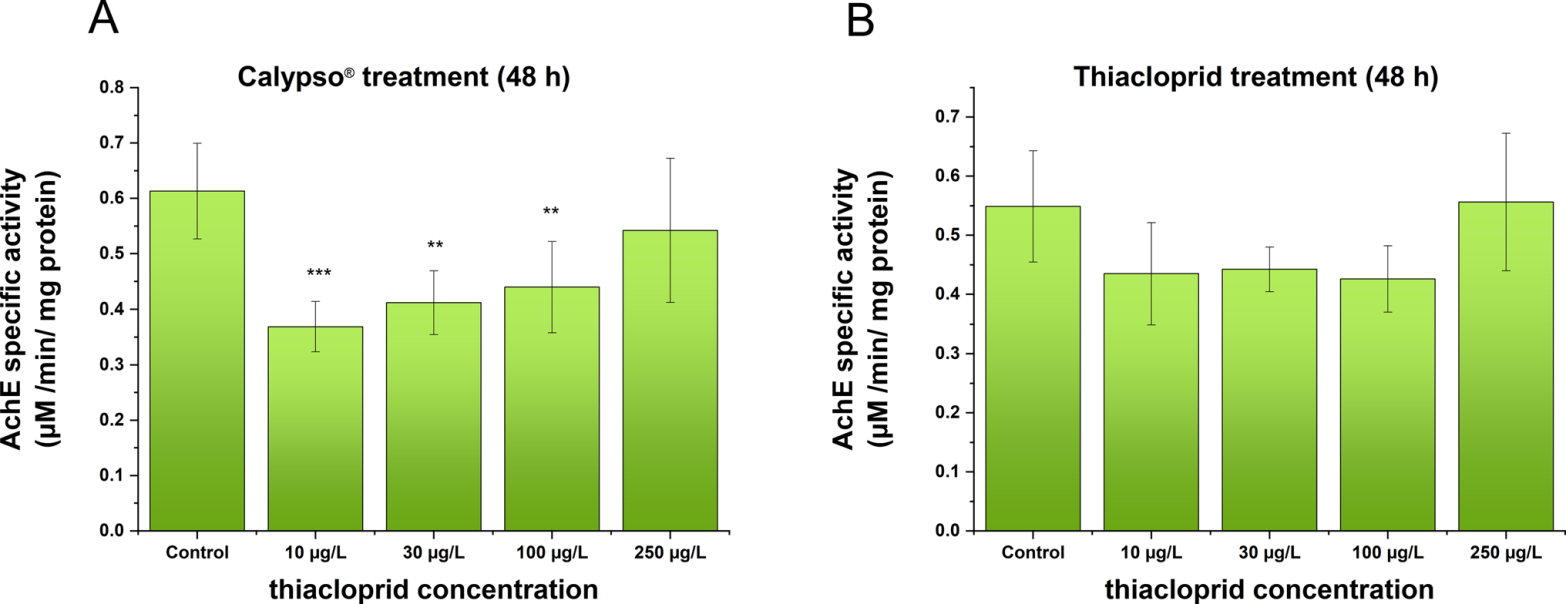
Alterations of AChE levels in *D. villosus* after Calypso® (**A**) and thiacloprid (**B**) treatments. Concentration values on the abscissa for Calypso® are nominal concentrations calculated from the composition of the formulation reported by the manufacturer, and for thiacloprid are nominal concentrations of the compound in its solutions applied in the test. Each bar represents the mean ± SD (*n* = 10 animals/group/replicate). Compared with the control, significantly decreased AChE activity was observed in the 10 µg/l (*p* < 0.001), 30 µg/l (*p* < 0.01) and 100 µg/l (*p* < 0.01) Calypso®-treated groups (**A**). The AChE activity decreased in all thiacloprid-treated groups except those treated with the highest concentration (**B**)

The alterations in AChE enzymatic activity are presented in Fig. 3. Compared with that in the control group, AChE activity decreased in all the Calypso®-exposed groups (Fig. 3A). Specifically, the highest inhibition was caused by the lowest Calypso® concentration (corresponding to 10 µg/l TIA), with a progressively lower inhibition potential at higher treatment concentrations, resembling a U-shaped plot of activity as a function of the concentration ratio. There was no significant difference in AchE levels in gammarids between the control and TIA-treated groups (Fig. 3B). However, AChE activity was lower in the 10 µg/l, 30 µg/l and 100 µg/l TIA-treated groups than in the control group, and a U-shaped change was detected.

## Discussion

Neonicotinoids impair cholinergic neurotransmission in the CNS but also affect several sublethal endpoints, including behavior, immobility and inhibition of feeding. It has been shown that nAChRs are targeted in the CNS of the pond snail *Lymnaea stagnalis* (Vehovszky et al. 2015) and behavioral and biochemical (AChE, GST) alterations are induced by acute CLO and IMI exposure in the killer shrimp *Dikerogammarus villosus* (Somogyvári et al. 2022). In the present study, we performed behavioral tests to investigate changes in immobility time (locomotion activity) following acute (2-day) exposure of *D. villosus* to TIA and Calypso®. Given that *D. villosus* is one of the most invasive freshwater species globally and has the potential to strongly impact local biota in novel areas, it might be beneficial to consider the wider implications of such investigations (Jermacz and Kobak 2018).

In terms of immobility time as an endpoint, there was a significant difference at a treatment concentration of 100 µg/l TIA compared with the control. The treated individuals were characterized by increasing inactivity up to this concentration. The group treated with the most concentrated solution (250 µg/l) presented activity comparable to that of the control group. Calypso® produced curves similar in shape to those of the pure agent. However, there was a significant change in the immobility time of individuals treated with Calypso® at concentrations as low as 30 µg/l, which reached 100 µg/l. Additionally, in the more concentrated solution (250 µg/l), the formulated neonicotinoid exhibited swimming activity comparable to that of the control group, i.e., the individuals displayed lively movement. It was shown that both the feeding rate and locomotor alterations were directly related to levels of AChE inhibition in *Gammarus fossarum* (Xuereb et al. 2009). Significant relationship between AChE activity levels and inhibition of behavior strength was reported in *Daphnia magna* as well (Ren et al. 2015). For all exposures, the trend of AChE activity was consistent with the behavioral responses of *D. villosus*, and no significant changes were found at the highest concentration compared to the control. This phenomenon may be in part explained by high concentration of neurotransmitters such as acetylcholine would suppress the synaptic transmission by an action at a presynaptic autoreceptor and activate its degrading AChE enzyme production, which results in such a dose–response curve (Calabrese 2008). Behavioral tests, such as locomotor activity, are sensitive and specific for detecting neurotoxic effects, as behavior is expected to be directly or indirectly related to the functioning of the nervous system. The effect of TIA on spontaneous tail coiling activity was tested, and TIA exposure of zebrafish larvae (≥ 50 μM with a 24 h exposure of the embryos to the medium) led to a reduction in the number of tail coils measured (Von Hellfeld et al. 2022; von Wyl et al. 2023). Muscle integrity was affected by TIA (concentration 125 mg/l, time of exposure 24 h) in zebrafish larvae (Könemann et al. 2022). TIA-induced swimming deficits (50–800 μg/ml) were reported in young worms, and swimming behavior was reduced (Scharpf et al. 2022).

In our experiments, sublethal effects were measured by means of GST and acetylcholinesterase enzyme activity. The class of GSTs enzymes are important contributors of the phase II metabolism of a broad range of xenobiotics, catalyzing their conjugation to glutathione (GSH) as ligand. The resultant metabolites having increased water solubility may be more easily eliminated from cells, thereby a reduced cell damage may occur. We observed a significant increase in GST activity upon exposure to Calypso®, suggesting the activation of the glutathione biotransformation pathway.

Our results showed that lower concentrations had a more significant effect than did higher concentrations. The GST activity first increased in the 10 and 30 µg/l Calypso®-treated groups. The significantly increased GST activity was observed only in these 2 groups, but at the highest concentration, the GST activity was not significantly different from that in the control group. The increased GST activity induced by exposure to Calypso*®* suggested metabolic activation in treated gammarids.

IMI-induced oxidative stress also elicited an increased GST activity in benthic bivalves at a 20 μg/l concentration (Shan et al. 2020). Enhanced GST activity after 24 h of exposure to Confidor® 200SL (a commercial formulation of IMI) was also reported in the amphipod *Gammarus fossarum* (Malev et al. 2012). In another study, IMI was tested on *Folsomia candida,* and it caused an increase in GST activity (Sillapawattana and Schäffer 2017). The activities of GST in the hemolymph of Sydney rock oysters, *Saccostrea glomerata*, were also significantly impacted, as reflected by the induction of GST activity after 4 days of exposure to environmentally relevant concentrations (at ≥ 0.1 mg/l) of IMI (Ewere et al. 2020). Elevated GST activity in the abdomen and hepatopancreas was also measured in tiger shrimp tissues (Butcherine et al. 2022).

To the best of our knowledge, the biotransformation of TIA in amphipods has not been previously reported in the literature. Among the four metabolites of IMI that were tested, IMI-ole, the metabolite that was identified as toxic in acute tests and defined as bioactive, was found to be readily biotransformed from the parent IMI. (Huang et al. 2021). The pyridinyl (A1) chlorine substituents are partially displaced by glutathione (Casida 2011), suggesting that GSH is the key to the increased GST activity caused by IMI (Somogyvári et al. 2022). It is likely that at higher concentrations (above 100 μg/l), the free GSH was already depleted during the 2-day treatment. The disappearance of GSH during treatment may lead to a slowdown in conjugate formation. Therefore, by the end of the treatment period, in the absence of GSH, the activity of GSH no longer changed. This finding was confirmed by studies in which the measurement of GST activity depended on the duration of neonicotinoid exposure (Radwan and Mohamed 2013; Stara et al. 2021).

At the biochemical level, changes in AChE enzyme activity were investigated in response to TIA and Calypso®. In contrast to previous observations for other pesticides, neonicotinoids do not directly inhibit AChE activity. This is probably because, unlike acetylcholine, neonicotinoids are not hydrolyzed by acetylcholinesterase. On the other hand, there is increasing evidence that AChE is a potential target of neonicotinoid action, as altered AChE activity has been reported in tissues of insecticide-treated animals of different taxa (Boily et al. 2013; Könemann et al. 2022). We also cannot exclude the possibility that AChE may interact directly with neonicotinoids (Győri et al. 2017).

We tested a possible indirect effect on *D. villosus* exposed to TIA and Calypso®. We observed a decrease in the activity of the AChE enzyme after the application of the commercial formulation. Significantly reduced AChE activity was observed at all the concentrations used. However, the change in AChE activity was not significant for the AI. In both cases, U-shaped enzyme inhibition was observed as a function of concentration. There are few data available in the literature concerning the effects of TIA on the cholinergic system, particularly on AChE activity. Similarly to our results, imidacloprid gave a U-shaped kinetics of the AChE activity with significant inhibition at the middle range (0.1 mg/l and 1 mg/l) in the tissues of the marine mussel (*Mytilus galloprovincialis* Lam.), but at the highest concentration (10 mg/l) the effect was not significantly different from the control. (Dondero et al. 2010). Acetylcholinesterase activities in the soft tissues of Sydney rock oyster to different concentrations (0 to 2 mg/l) of imidacloprid was measured. Unexpected dose effects revealed no evident correlation between dosage and the activity of gill AChE. AChE activities of oysters exposed to lower concentration (1 mg/l) were significantly lower from the control; however, exposure to the highest concentration (2 mg/l) of imidacloprid did not significantly inhibit gill AChE. It has been hypothesized that this may be due to the metabolic activity of the pesticides at highest concentrations, or to a complex interplay involving gene reprogramming effects and receptor binding (Ewere et al. 2019a). The highest concentration applied, 10 mg/kg Calypso®, caused a decrease in AChE activity in the earthworm *Eisenia andrei*, and 1 mg/kg Calypso® also caused a decrease in AChE activity in the same work (Lackmann et al. 2023), while resulted in AChE activation along with reduced reproduction rates (Lončarić et al. 2024). For nontarget aquatic organisms, reduced AChE activity following IMI exposure has been reported in shrimp (Butcherine et al. 2022), oysters (Ewere et al. 2019b) and zebrafish (Guerra et al. 2021). Our data appear to be of environmental relevance for TIA, as this effective dose is now close to the TIA detection value measured in surface waters reported worldwide (Morrissey et al. 2015), where the highest concentration (12 µg/l) was measured residing in surface waters of The Netherland (Barmentlo et al. 2018).

The role of adjuvants in insecticide formulations in modifying toxicity is extremely important, and related toxicity problems should be considered in the authorization of plant protection products (Klátyik et al. 2017). Some additive changes the physicochemical properties of the AI (e.g., increase solubility in water) or enhances uptake into the test organism. In our earlier publication (Takács et al. 2017), we have studied the acute toxicities of three neonicotinoid containing formulations on *Daphnia magna*, and compared to that of their AIs. Apache® 50 WG proved to be 46.5 times more toxic for the applied aquatic test organism, whereas toxicity of Actara® 240 SC and Calypso® 480 SC was less toxic, than their corresponding AIs CLO, TMX and TIA, respectively. Individual and combined toxicity was also studied with another neonicotinoid containing formulation, Mospilan® 20 SG, including ACE as an AI and linear alkylbenzene sulfonates (LAS) formulating agent (Mörtl et al. 2020). Individual toxicity of LASs was significantly higher on *D. magna* in acute immobilization test than that of ACE. Synergistic toxicity was found, when pure forms of the investigated components (LAS and ACE) were used in combination, and somewhat lower synergy has been observed for Mospilan® 20 SG. GST enzyme activity assays were also performed to investigate the individual and combined toxicity. ACE individually or in combination with LASs reduced the GST enzyme activity, whereas no significant differences were observed between the control group and those exposed to LAS alone or to Mospilan®. Thus, in contrast to the immobilization tests, high toxicity of LASs was not detected on GST activity. As the previous examples also demonstrate, the co-occurrence of the AIs and surfactant may significantly alter the effect the AIs on nontarget species, but direction depends on the test used and the investigated endpoint as well. Pesticide formulating agents (e.g., surfactants) may have direct toxic effects. A well-known example was reported for glyphosate formulations containing polyethoxylated tallow amine (POEA), which is not an inert ingredient, and it has much higher toxicity to terrestrial (Klátyik et al. 2023) as well as aquatic species (Klátyik et al. 2024) than glyphosate itself. Determination of the own adverse effects of surfactant is usually not so simple as safety data sheets (SDS) do not provide detailed information about the molecular characteristics of the additive or composition of commercial mixtures are very complex (e.g., homologue distribution) or even variable (Tush et al. 2013). Lack of analytical standards for the quantification or ecotoxicological testing also prevents the precise characterization and risk assessment. Regarding the components of Calypso®, it contains 10% of glycerine (Bayer SDS), which is a nontoxic substance, used in cosmetics as well. Moderate water solubility (185 mg/l at 20 °C) of TIA is significantly increased to 480 g/l in the formulation, which may result in enhanced uptake into the test organism. Benzisothiazolinone, a widely used preservative, is also listed among the components of Calypso®. This chemical has a microbicide and fungicide effect, and according to regulations in the EU, it is not allowed to use in cosmetics. No other surfactant or additive is indicated on the SDS (Bayer SDS).

The present study suggests that the presence of the adjuvant in exposure media enhanced the alteration of TIA on the behavioral and cellular traits considered. These results underline the need to further clarify the mechanisms that determine the increased biological activity of TIA in its formulation studied as compared to its effects exerted alone.

## Conclusion

This study demonstrated that Calypso® caused significantly stronger impairments in locomotor activity and cellular alterations in *D. villosus* at doses containing equivalent concentrations of AI as the pure TIA. This suggests that the formulant present in the product increased its toxicity potential in this aquatic invertebrate. The alterations in locomotion and in cellular traits exhibited a similar pattern, with significantly higher intensity characteristic of Calypso®. Our results indicated that acute exposure to Calypso® or TIA, even at sublethal concentrations, has the potential to adversely impact the survival rate of this particular amphipod species. This may consequently lead to deleterious consequences on a population level over an extended time. Our results underscore the necessity to identify the pathway of joint toxic action of the adjuvant(s) in Calypso® and the AI, which could be of high relevance for the proper evaluation of the aquatic toxicity risks of TIA and TIA-based agrochemical formulations.

## Data Availability

The datasets analyzed during the current study are available from the corresponding author on reasonable request.
